# Directional radiation of Babinet-inverted optical nanoantenna integrated with plasmonic waveguide

**DOI:** 10.1038/srep11832

**Published:** 2015-07-02

**Authors:** Jineun Kim, Young-Geun Roh, Sangmo Cheon, Un Jeong Kim, Sung Woo Hwang, Yeonsang Park, Chang-Won Lee

**Affiliations:** 1 Samsung Advanced Institute of Technology, 130, Samsung-ro, Yeongtong-gu, Suwon-si, Gyeonggi-do, 443-803, Korea; 2 Center for Artificial Low Dimensional Electronic Systems of the Institute for Basic Science, 77, Cheongnam-Ro, Pohang, 790 - 784, Korea

## Abstract

We present a Babinet-inverted optical nanoantenna integrated with a plasmonic waveguide. Using an integrated nanoantenna, we can couple the plasmon guide mode in a metal-insulator-metal (MIM) structure into the resonant antenna feed directly. The resonantly excited feed slot then radiates to free space and generates a magnetic dipole-like far-field pattern. The coupling efficiency of the integrated nanoantenna is calculated as being approximately 19% using a three-dimensional finite-difference time-domain (3D FDTD) simulation. By adding an auxiliary groove structure along with the feed, the radiation direction can be controlled similar to an optical Yagi-Uda antenna. We also determine, both theoretically and experimentally, that groove depth plays a significant role to function groove structure as a reflector or a director. The demonstrated Babinet-inverted optical nanoantenna integrated with a plasmonic waveguide can be used as a “plasmonic via” in plasmonic nanocircuits.

The optical nanoantenna has been proven to be an important tool for the control of electromagnetic (EM) waves in the visible or near-infrared regions, below the diffraction limit^1^,^2^,^3^. Using this device, we can efficiently detect resonant light and radiate it in a dipole-like pattern in the sub-wavelength scale. In particular, the optical counterpart of the Yagi-Uda antenna also demonstrates radiation routing to a certain direction, which enables successful electromagnetic energy transfer from one point to another^4^,^5^,^6^. Therefore, considerable research into harnessing the functionalities of optical antennae with integrated optical nanocircuits has been conducted^7^. For example, bow-tie antenna-integrated Ag nanowires^8^, rod-arrayed antennae with dielectric waveguides^9^, nanoparticle antennae with dielectric waveguides^10^, bidirectional plasmonic antennae for color routing with dielectric waveguides^11^,^12^, Yagi-Uda antennae with gap surface plasmon (SP) guides^13^, waveguide-loaded plasmonic antennae for magnetic storage devices^14^, and slot-type antennae with electrical-driven plasmon sources^15^ have been demonstrated recently.

Most of the reported research uses the rod-type optical nanoantenna. For plasmon resonance, this nanoantenna should be isolated from contact with metal film and passivated by an insulating region. If it is attached to other plasmonic components made of metal, the resonance condition collapses and the nanoantenna does not work. Recently, we reported that a metallic slot structure can be regarded as a magnetic dipole and functions as a Babinet-inverted optical nanoantenna^16^. We also proved that a Babinet-inverted Yagi-Uda nanoantenna composed of a feed, reflector, and director can control the direction of resonant light, like the rod-type Yagi-Uda nanoantenna. In the case of the Babinet-inverted nanoantenna, all components are made on a metallic film. Therefore, any plasmonic components positioned on the metal layer can be fabricated in any place containing metal film, apart from the optical nanoantenna area. Thus, the Babinet-inverted optical nanoantenna has high potential for integration and combination with other plasmonic components. Here, we present a Babinet-inverted optical nanoantenna integrated with a metal-insulator-metal (MIM) plasmonic waveguide. We experimentally demonstrate that the plasmon guide mode of the MIM structure is coupled to a magnetic dipole (Babinet-inverted) feed, depending on the slot direction at the resonant frequency, and radiates to free space in a dipole-like pattern.

Figure 1 shows the concept and schematics of the Babinet-inverted optical nanoantenna integrated with a MIM plasmonic waveguide. A long slit at the bottom layer of the MIM waveguide launches a surface plasmon (SP) wave from the incident laser light with perpendicular polarization to the slit^17^,^18^. The launched SP wave propagates normal to the slit and forms the transverse magnetic (TM) mode. If the slot direction is not perpendicular to the slit, the SP wave can induce a magnetic dipole moment in the slot, which acts as a local dipole source at the top surface and radiates to free space. Additionally, we add an auxiliary shallow groove, working either as a reflector or as a director depending on the depth. The resultant antenna consists of a magnetic dipole feed generating free-space radiation and a groove element generating out-of-phase response. The Babinet-inverted optical Yagi-Uda antenna integrated with the MIM plasmonic guide couples the propagating plasmon guide mode into a resonant antenna feed mode and exhibits radiation with a certain degree of directionality.

Our integrated antenna based on the magnetic dipole feed has several advantages over an electric-dipole-based structure. First, it has a large signal-to-noise ratio because of its metallic layer, which naturally creates two regions between the front and back layer. When incident light is launched from the back layer, the metallic layer blocks it, while measuring the scattered light from the front layer. Therefore, the Babinet-inverted antenna shows a larger signal-to-noise ratio compared with a rod-type antenna without using a blocking element. Second, the Babinet-inverted antenna plays the role of a vertical coupler. The SP mode propagating laterally through the MIM structure is coupled into the slot feed, and a slot feed makes vertical radiation with a dipole-like pattern. This means that a Babinet-inverted antenna integrated with MIM plasmonic guide changes a lateral-guided mode to vertical radiation. If radiation is launched into a slot, it is coupled to lateral-propagating guided mode by the help of a slot feed. Therefore, we can regard a slot feed as vertical coupler located on MIM plasmonic guide. Third, the combination of the magnetic antenna and the MIM waveguide provides a potentially feasible bias application between two metal slabs to enable electrical excitation of a dipole. Recently, subwavelength optical nanocircuits were electrically driven using a MIM-gap SP guide structure^15^. Finally, the integrated Babinet-inverted antenna acts as a “plasmonic via” in three-dimensional nanophotonic circuits composed of several layers. A Babinet-inverted antenna has a role of coupling lateral guiding mode to vertical radiation, and plasmonic connection between physical layers in 3D photonic circuits that goes through the plane of one or more adjacent layers. It is an exact counterpart of the “electrical via” in electronic circuits.

## Results and Discussion

### Mode coupling between MIM plasmon-guide and single slot

**Figure 2:**
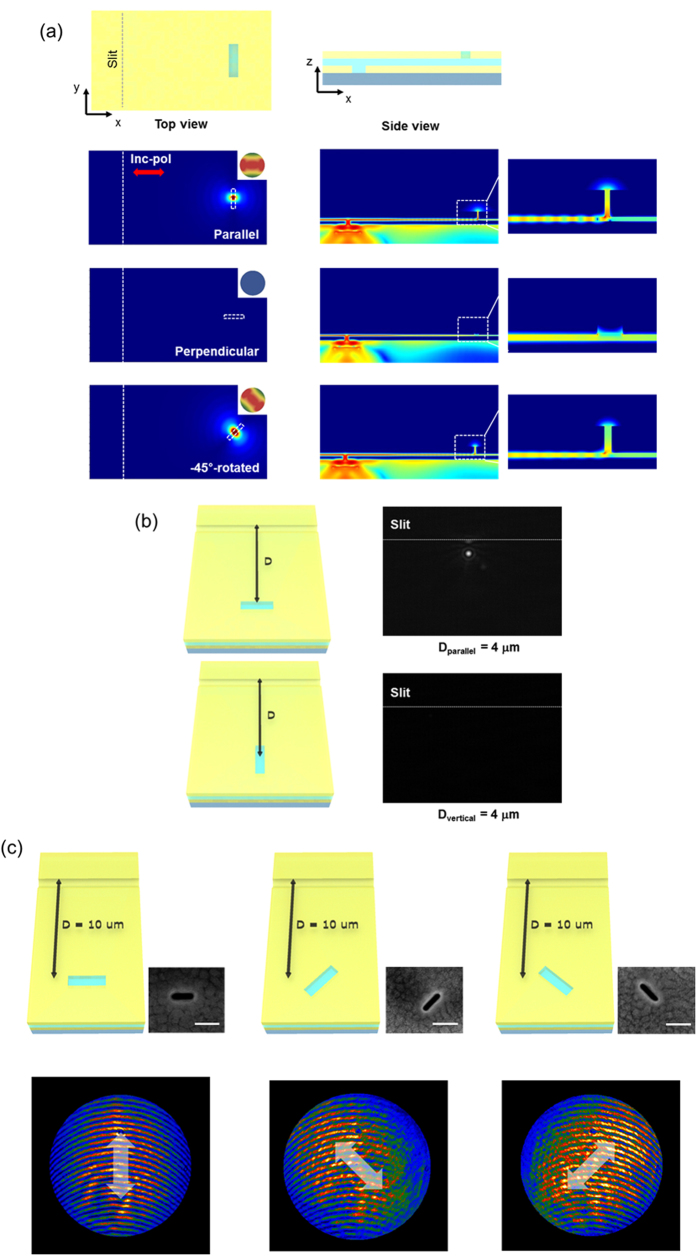
Mode coupling between MIM plasmonic waveguide and single slot. (**a**) Results of 3D FDTD simulation. The first column represents the *x*-*y* cutting plane of the simulation (The z-position is 60 nm from top metal surface.), where the slit and slot are represented by a dotted line. The red arrow shows the direction of the incident polarization. The second column represents the *x*-*z* cutting plane (The y-position is the center of slot.). Inset of column the first shows far-field radiation pattern evaluated from simulation. The third column shows magnified side view of slot area. The red-blue contour map shows the intensity of electric field. In the case of the single slot parallel to the slit (second line of figures), we can see that the plasmon is coupled to the single slot. However, the plasmon is not coupled to the slot perpendicular to the slit (third line of figures). From the far-field results incorporating a −45°-rotated single slot (fourth line of figures), we see clearly that the direction of radiation is vertical to the slot. (**b**) The measured outcoupled light images and the schematic geometry of the fabricated samples are shown. The captured images of the transmitted light in the sample with 4 μm distance are shown in the right column of figures. (The results with other distance are shown in Fig. S2(a).) We observe bright coupled light in the parallel slot sample only. (**c**) The first line of figures shows the schematic geometry and SEM image of the sample used for the Fourier-space image experiment. The white line in the SEM was 200 nm in length and single slots with three orientations were fabricated. One was oriented parallel to the slit, and the others were 45°- and −45°-rotated. The distance between the slit and a single slot was 10 μm. The Fourier-space images of the transmitted light are shown in the bottom line of figures and the direction of the dipole-like radiation is vertical to the slot. For ease of observation, white thick arrows representing the direction of radiation were drawn in the Fourier-space images.

We used the three-dimensional finite-difference time-domain (3D FDTD) method to simulate the SP wave propagation, coupling, and radiation from the magnetic antenna, as shown in Fig. 2(a)^19^. The structure of MIM plasmonic guide is made of silver(Ag)-poly(methyl methacrylate)(PMMA)-Ag materials. While doing FDTD simulation, we used parameters same to those of experiment. The thickness of bottom Ag layer, guiding PMMA layer, and top Ag layer are 180 nm, 50 nm, and 300 nm. The slit width corresponds to 135 nm as like real sample. The index data of silver metal are adapted from Palik’s data^20^, and those of the PMMA come from ref. 21. The Gaussian beam is excited in FDTD simulation to resemble with experimental source of 664 nm laser with around 10 μm spot size. The schematics are represented in the first line of Fig. 2(a) and the long slit formed in the bottom metal layer is represented by a dotted white line. The distance between the slit and slot was 5 μm. The simulation shots comprising the left column of the figure show an *x*-*y* plane (top view) of the 3D simulation model, while the right column shots are in the *x*-*z* plane (side view). The simulation contains single slots with three different directions: parallel (second line), vertical (third line), and 45°-rotated (fourth line) relative to the slit direction. In simulation, sources with an electric field (E-field) perpendicular to the long side of the slit (the *x*-direction in Fig. 2(a)) were launched from the substrate. The contour image map in the simulation cutting plane corresponds to the E-field intensity directly. We see in the *x*-*z* plane that the incident light becomes a plasmon under the effects of the slit structure, and that the generated plasmon is well-guided through the MIM structure. The simulation shows that the direction of the single slot on the top metal layer determines the coupling between the antenna and guide modes. In the case of a vertical slot (third line in Fig. 2(a)), the plasmon in the MIM waveguide does not couple to the slot mode and propagates as if there is no structure. In contrast, the plasmon strongly couples to the resonant mode of the single slot if the slot is parallel-oriented to the slit (second line in Fig. 2(a)). In this case, the slot functions as an antenna radiating to free space. We also observe that the near fields from the slot are vertical to the long-direction of the single slot. This means that the near-field pattern is identical to that of a magnetic dipole with the same direction as the single slot. To confirm the near-field orientation, a 45°-rotated single slot was also simulated (fourth line in Fig. 2(a)). As like changing on the direction of near-field depending on the slot orientation, direction of far-field radiation is also changed. Using near-field distribution of simulation result, far-field transformation is executed (third column of Fig. 2(a)). From the near- and far-field simulation results, we can see that the radiation direction varies depending on the orientation of the single slot.

To investigate the coupling conditions experimentally, we prepared several slot samples with different orientations and distances from the SP launching slit. The sample geometry is shown in Fig. 2(b), schematically, and it can be seen that each slot has two kinds of orientation, *i.e*., parallel and vertical to the slit direction. Six sets composed of two slots were fabricated using focused-ion-beam (FIB) milling, with distances from the slit of 1, 2, 4, 6, 8, and 10 μm. Here, each slot was isolated from the other by a distance of 10 μm so as not to interrupt the measurement. A laser with a 664-nm wavelength was used in the coupling measurement experiment, and the polarization of the incident light was modulated by a polarization filter placed before the sample, while the transmitted image was captured by an electron-multiplying charge-coupled device (emCCD) on the front side of the sample. The length of a single slot was set to 180 nm so as to match the resonance condition at 664 nm. In Fig. 2(b), we see that a single slot parallel to the slit shows transmission light, but the slot vertical to the slit does not (Supplementary Information 2). These findings are very consistent with the results of the 3D FDTD simulation shown in Fig. 2(a). To confirm the coupling between the plasmon guide mode and single-slot resonant mode, we rotated the incident polarization direction. Even for a slot parallel to the slit, if the incident light polarization was perpendicular to the slit, we did not observe a transmitted intensity because the plasmon was not generated in the slit position. From these experimental results, we conclude that the guided plasmon is coupled to the resonant mode of the slot antenna (Supplementary Information 3).

As additional evidence of coupling, we examined Fourier-space images of the transmitted light (Supplementary Information 4 & 5). If the plasmon is well-guided through the MIM structure and coupled to the antenna resonant mode, the measured light of Fig. 2(b) should come from a single-slot antenna and should have a dipole-like shape, as reported in ref. 16. We also prepared a MIM-integrated slot antenna with three orientations for the Fourier-space image measurement, and the schematic geometry is given in the upper line of Fig. 2(c). The first single slot had a direction parallel to the long slit, while the orientations of the second and third slits were 45°-rotated and -45°-rotated, respectively. The distance between the slit and slot was 10 μm, and each slot was sufficiently isolated (by 30 μm) to not be affected when the Fourier-space image was obtained. SEM images of the fabricated samples are shown in Fig. 2(c). Each Fourier-space image shows a dipole-like far-field radiation pattern perpendicular to the long side of slot and we can therefore see experimentally that the orientation of the radiation from the slot changes depending on the slot direction orientation (white arrow in the second line of Fig. 2(c)). Note that the line pattern in the Fourier-space image comes from the slit structure and a minor focusing mismatch. These transmission and Fourier-image measurements confirm the coupling between the plasmon guide mode and the single slot antenna resonant mode.

**Figure 3:**
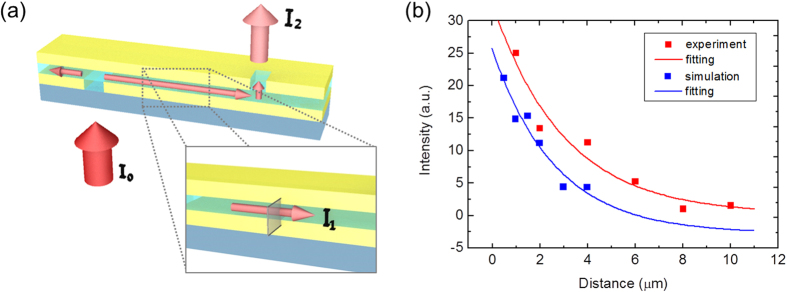
Model of coupling efficiency calculation and propagation length of MIM plasmonic waveguide. (**a**) Model of analytic calculation of coupling efficiency. *I*_*0*_ represents the incident light power, *I*_*1*_ the plasmon guide mode power at the slot incident position (100 nm apart from the antenna), and *I*_*2*_ corresponds to the final output power radiating from the slot antenna (100 nm apart from the antenna in z-axis). (**b**) Output intensities as functions of propagating distance are plotted and fitted as a first-order exponential decay function. The blue (red) rectangles show the intensities obtained from the 3D FDTD simulation (experiment) and both the blue and red lines are the fitted graphs. The propagation length of the MIM plasmonic waveguide is approximately 3 μm.

In a plasmon-guide-integrated optical antenna, the amount of incident power that is transformed into nanoantenna radiation is an important factor as regards real application. We define the ratio of radiation power to incident guided-plasmon power as the efficiency of the plasmon-guide-integrated optical antenna (Supplementary Information 6). In our experiment, when light was launched into the long slit, it was changed into a plasmon and delivered to the single slot antenna through the MIM structure. The plasmon coupled to the guide mode decays exponentially during propagation in the MIM structure and, at the nanoantenna position, the plasmon guide mode is coupled to the antenna resonant mode and radiates as light to free space. Therefore, the coupling efficiency of the plasmon-guide-integrated antenna (C) is regarded as 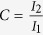
(where *I*_*1*_ is the incident guided-plasmon power and *I*_*2*_ is the transmitted power, Fig. 3(a)).

In this letter, we conducted a 3D FDTD simulation and calculated the coupling efficiency from the simulation results directly (Supplementary Information 6). Simulations for the cases with slit-slot distances of 1, 2, 4, 6, 8, and 10 μm were conducted under the same conditions as the physical experiment, and the radiation power from the antenna was calculated. The calculated coupling efficiency was found to be approximately 19%, similar to that of the rod-type antenna reported in 2012^9^. Figure 3(b) shows the exponential decay of the plasmon propagation. By fitting the graph to a first-order exponential function, we determined that the MIM plasmon guide structure had a propagation length of approximately 3 μm^22^,^23^ (simulation results: blue rectangles and experimental data: red rectangles) (Supplementary Information 2). This supports our view that 3D FDTD simulation is a good approach to determining the coupling efficiency of a plasmon-guide-integrated nanoantenna.

### Steering the direction of far-field radiation by Babinet-inverted antenna integrated with plasmonic waveguide

#### Simulation for reflector and director groove structure conditions

In 2010, Kosako *et al.* demonstrated that an optical Yagi-Uda nanoantenna, with auxiliary elements known as the reflector and director, can control the direction of far-field radiation, even in the optical regime^4^. After that, unidirectional optical nanoantennas by plasmonic nanodisk^24^ and broadband unidirectional scattering by magneto-electric core-shell nanoparticles are developed^25^. In case of Yagi-Uda type nanoantenna with unidirectionality, the auxiliary elements, located in the proper position relative to the feed, reflect or direct radiation. Therefore, we can ‘steer’ the far-field radiation in particular directions using these auxiliary elements. Previously, we have experimentally proven that a Babinet-inverted optical antenna composed of all slot-type elements can also produce unidirectional radiation, like the rod-type Yagi-Uda nanoantenna^16^. However, in this letter, we do not choose a slot structure as an auxiliary element, but instead use a groove structure so as to overcome the loss problem. That is, the slot feed plays two roles, capturing the guided plasmon with a wavelength resonant to the metallic slot and radiating the resonant mode into the far-field radiation pattern. If other slot structures are located in the same plane as the feed, it will act as another capturing point for the guided plasmon, even though the efficiency is very low because of the lack of resonance and, as a result, the nanoantenna efficiency will be decreased. To prevent loss by another element, a groove structure is adapted as an auxiliary element in this experiment. To determine if this groove structure will also act as a reflector or director, we performed both a 3D FDTD simulation and a practical experiment with various groove conditions. Hence, we determined the conditions under which the groove structure functions as a reflector or director.

**Figure 4:**
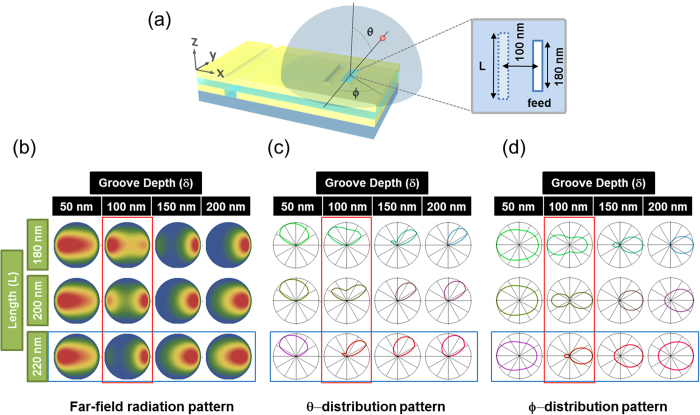
Model configurations for near-to-far-field transformations and matrix map of simulated far-field pattern. (**a**) Geometrical configurations of near-to-far-field transformation simulation. The hemisphere is cut by the *x*-*z* (vertical) and *x*-*y* (lateral) plane and each cutting plane corresponds to a θ- and *ϕ*-distribution of the far-field radiation. The structure composed of a single slot and groove is located at the origin of the hemisphere. The groove structure length is labeled *L* and the distance between the feed and groove is fixed as 100 nm. (**b**) Far-field radiation pattern calculated from near-to-far field transformation. (**c**) θ-distribution of simulated far-field radiation. (**d**) *ϕ*-distribution of simulated far-field radiation. The red box represents the role change (from weak director to reflector as the length increases) of the groove, depending on its length at fixed depth. The blue box represents the role change (from director to reflector as depth increases) of the groove, depending on its depth at fixed length.

We considered two geometric parameters of the groove structure, length and depth as a controlled parameter because the effect of the distance from the previous work turns out to be smaller^16^. Therefore, we fixed the distance between the feed and the auxiliary element as 100 nm during the simulation. The feed length was fixed at 180 nm, resonant to the wavelength of 664 nm (Fig. 4(a)) and we changed the groove length (L) from 180 to 220 nm in 20-nm increments, and the groove depth (δ) from 50 to 200 nm in 50–nm steps. In total, 12 groove condition setups were simulated and evaluated. To determine the appropriate reflector and director conditions at the resonance frequency, a near-to-far-field transformation method was used and far-field radiation patterns for each condition were visualized in table form. The far-field radiation pattern was obtained by evaluating the total fields by 1 m away from the point, where the antenna and the source were located. By projecting the three-dimensional field distribution on the hemi-sphere into a two-dimensional circle plane, we obtained the far-field radiation pattern shown in Fig. 4(b). For the Yagi-Uda antenna, an auxiliary element shorter than that of the feed works as a director, while an auxiliary element longer than the feed generally functions as a reflector^26^,^27^. Given a fixed groove depth (δ) with 100 nm, we see this trend in the calculated results (red box in Fig. 4(b–d)). Additionally, we also found that the shallow-depth grooves can also play the role of directors, even in long-length conditions since, as the groove depth increases, the groove changes from functioning as a director to a reflector (blue box in Fig. 4(b–d)). To see this effect more clearly, radiation patterns of *θ*-distribution (cut by the *x*-*z* plane) and *ϕ*-distribution (cut by the *x*-*y* plane) are shown in Fig. 4(c,d) respectively.

#### Directional radiation from groove structure

**Figure 5:**
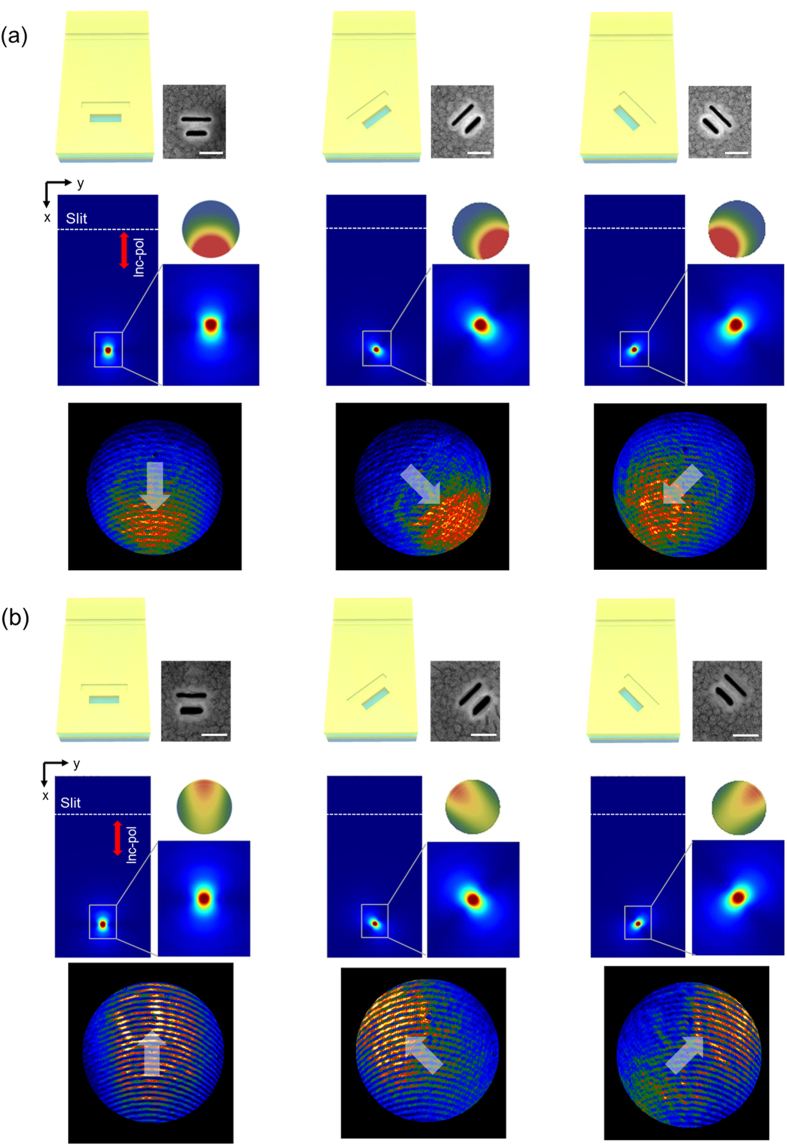
Directional radiation from slot and groove structure. (a) Simulated and experimental results of groove reflector sample. The groove structure has a depth of 100 nm, distance of 100 nm from slot antenna, and length of 280 nm. It plays the role of a reflector, according to the results of Fig.4 (b–d). The slit is represented by the dotted line and the combined slot and groove structure is located 5 μm from the slit position. The first line of figures shows the schematics and SEM images of the samples with parallel, 45°-rotated, and −45°-rotated orientations with respect to the slit, while the second line of figures gives the simulated 3D FDTD results and far-field radiation patterns. The red-blue contour image map in the *x*-*y* plane shows the intensity of electric field, while the measured Fourier-space images are shown in the third line of figures. We see clearly that the direction of far-field radiation from the slot and groove is opposite to that of the region where the groove is located, and changes depending on the orientation of the combined structure. This means that the groove acts as reflector. For ease of observation, thick white arrows have been drawn on the measured Fourier-space images. (**b**) Simulated and experimental results of groove director sample. The groove structure has a depth of 50 nm, distance of 120 nm from slot antenna, and length of 280 nm. It plays the role of a director according to the results of Fig.4 (b–d). The first line of figures shows the schematics and SEM images of the samples, while the second line represents the simulated near- and far-field pattern from 3D FDTD. The measured Fourier-space images are shown in the third line of figures. We see clearly that the direction of the radiation from the slot and groove is toward the region where the groove is located, and changes depending on the orientation of the combined structure, indicating that the groove acts as a director. For ease of observation, thick white arrows have been drawn in the measured Fourier-space images.

To experimentally confirm the effect of the groove depth on the unidirectional control of the integrated Babinet-inverted nanoantenna, we conducted a transmission and Fourier-space image experiment similar to the dipole coupling measurement. We fabricated samples with two groove depths of 50 nm and 100 nm, as in the simulation. To create samples with groove structures of two different depths, the FIB milling process was precisely tuned by inspecting the groove depth in each beam condition (Supplementary Information 1). According to the near-to-far field transformation results (Supplementary Information 7), we expect that a groove of 100-nm depth should function as a reflector. To confirm this behavior, three antenna orientations were produced and examined, as in the Fourier-space dipole-coupling image experiment case, being normal, 45°-rotated, and −45°-rotated with respect to the slit. The upper line of Fig. 5(a) gives the schematics and SEM images of each sample, while the central line shows the *x*-*y* (lateral) plane on the surface of the top metal layer obtained by the 3D FDTD simulation. The red-blue contour image map represents the *x*-polarized E-field intensity directly, while the slit position is represented by a white dotted line. A multi-element structure composed of a single slot and single groove was located 5 μm away from the slit position.

In order to observe the direction of the radiation from the nanoantenna clearly, a near-field and far-field image maps of the radiation were amplified. In the amplified far-field inset of Fig. 5(a), we see that the light is steered downward clearly. This means that the groove structure with 100 nm depth reflects the radiation from the feed. We see this reflecting effect more clearly in the measured Fourier-space image shown in the bottom line of Fig. 5(a). A maximum front-to-back ratio (FB ratio) of 7.38 (8.68 dB) in the 45°-rotated groove reflector structure was obtained here^19^. For the case with the groove of 50-nm depth, it is apparent that it plays the role of a director in Fig. 5(b), as the near-field contour image map shows that the radiation is directed upward. However, because the influence of the director is very weak, contrary to the reflector case, we obtained a small maximum FB ratio of 3.01 (4.79 dB) for the 45°-rotated groove director structure. The direction of the far-field radiation is represented by the white arrow in the bottom line of Fig. 5(a,b) for clarity.

These calculated and experimental results confirm that the plasmon waveguide mode formed in the MIM structure is well-coupled to the Babinet-inverted nanoantenna containing both a slot and groove, similar to the single–slot case, and the radiation from the slot can be steered due to the groove effect, like the Yagi-Uda nanoantenna. Based on these results, it is apparent that we can efficiently control the direction of the radiation from the Babinet-inverted optical nanoantenna integrated with a plasmon waveguide.

## Conclusion

In conclusion, we have experimentally demonstrated a Babinet-inverted optical nanoantenna integrated with a plasmon waveguide. We showed that the plasmon guide mode of the MIM structure is well-coupled to the Babinet-inverted antenna composed of a single slot, depending on the slot direction, and it radiates with a magnetic dipole radiation shape. The coupling efficiency of the guide-integrated Babinet-inverted nanoantenna is calculated to be 19%, according to the 3D FDTD simulation. Additionally, by adding a groove structure to the slot-type feed on the top metal layer of the MIM structure, we can steer the radiation from the slot in a manner similar to a Yagi-Uda antenna. While searching for the appropriate groove structure conditions necessary to create a reflector using near-to-far field transformations, we determined that the groove structure acts as a either a reflector or director depending on its depth. We confirmed the effect of groove depth and demonstrated the directional control of the plasmon-guide-integrated Babinet-inverted antenna experimentally by preparing samples containing slots and grooves and measuring the Fourier-space image of the resultant transmitted light. In this experiment, we achieved a maximum FB ratio of 7.38 (8.68 dB) for the slot and groove reflector structure.

The Babinet-inverted optical nanoantenna integrated with a plasmon waveguide suggested and demonstrated in this letter can function as a “plasmonic via” in integrated plasmonic nanocircuits because of its spatial separation by metallic layer. As the slot-type antenna can be combined with any plasmonic components made on a metallic plane easily, we expect that the Babinet-inverted nanoantenna will be integrated further with other plasmonic devices and ultimately play an important role in plasmonic integrated nanocircuits in the future.

## Methods

### Sample fabrication

A 180-nm-thick Ag layer is deposited by electron beam evaporator on a double-sided polished glass substrate. A long slit of 100 μm in length and 135 nm in width is milled by a focused ion beam (FIB) using FEI Helios NanoLab. A dielectric layer composed of a mixture of poly(methyl methacrylate) (PMMA) is spun-coat at 3,000 rpm for 40 s and forms a plasmon guiding layer with 50-nm thickness. The resultant layer ensures the formation of a plasmonic guide mode, as opposed to a photonic guide mode. Then, a 300-nm-thick Ag layer is deposited on the PMMA layer to create the MIM structure. Slot antennae are also fabricated using FIB, and the slot depth is gently tuned to stop at the bottom interface of the top metal layer.

### Optical Measurement

To confirm coupling between the plasmon waveguide mode and the resonant mode of a single slot, we adapted the transmission measurement geometry generally used in plasmon-guide experiments. In the transmission measurement experiment, a 664-nm laser light with a Glan-Taylor polarizer was launched from the substrate side and focused on the slit position using an objective lens. A plasmon was generated by the slit structure formed in the bottom metal layer, which was then guided through the thin dielectric film and coupled to the resonant mode of the single-slot antenna. Because the single slot acts as a magnetic dipole, it radiates light into free-space and creates a dipole-like far-field pattern. At the front side of the sample, we can observe radiated light (detected by the emCCD camera) and also obtain a far-field radiation pattern using the Fourier-space image measurement setup (Supplementary Information 4).

## Additional Information

**How to cite this article**: Kim, J. *et al.* Directional radiation of Babinet-inverted optical nanoantenna integrated with plasmonic waveguide. *Sci. Rep.* **5**, 11832; doi: 10.1038/srep11832 (2015).

## Supplementary Material

Supplementary Information

Supplementary Movie

## Figures and Tables

**Figure 1:**
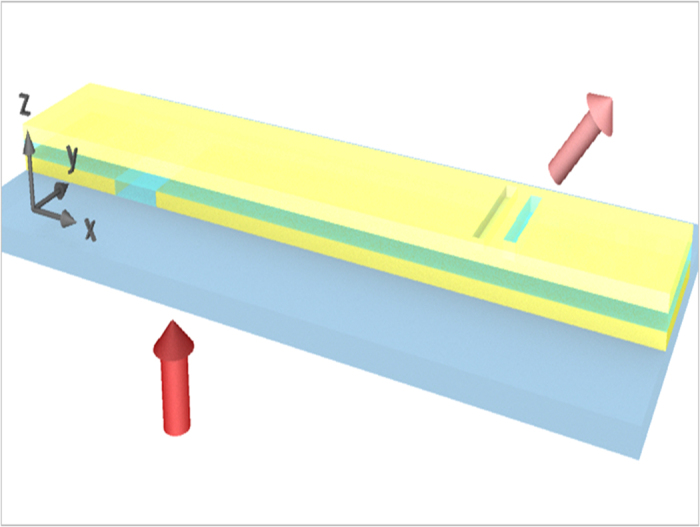
Babinet-inverted optical nanoantenna integrated with plasmonic waveguide schematics. Schematic figure of Babinet-inverted optical nanoantenna integrated with metal-insulator-metal plasmonic waveguide. The red arrows represent incident light launched from the glass substrate side of the sample and light radiating from the slot antenna to free space.
