# Supplementary material for: Directional radiation of Babinet-inverted optical nanoantenna integrated with plasmonic waveguide
Source: Sci Rep. 2015 Jul 2;5:11832. doi: 10.1038/srep11832 (PMC4488836; doi:10.1038/srep11832)
Supplement: Supplementary Information [file srep11832-s1.doc]

**Supplementary Information**

**Directional radiation of Babinet-inverted optical nanoantenna integrated with plasmonic waveguide**

Jineun Kim^1^†, Young-Geun Roh^1^†, Sangmo Cheon^1,2^, Un Jeong Kim^1^, Sung Woo Hwang^1^, Yeonsang Park^1^*, Chang-Won Lee^1^*

*^1^Samsung Advanced Institute of Technology, 130, Samsung-ro, Yeongtong-gu, Suwon-si, Gyeonggi-do, 443-803, Korea*

*^2^Center for Artificial Low Dimensional Electronic Systems of the Institute*

*for Basic Science, 77, Cheongnam-Ro, Pohang, 790 - 784, Korea*

†These authors contributed equally to this work.

Correspondence and requests for materials should be addressed to Y.P. (email: [yeonsang.park@samsung.com](mailto:yeonsang.park@samsung.com)) and C.-W.L (email: [chang-won.lee@samsung.com](mailto:chang-won.lee@samsung.com)).

**1. Sample fabrication**

The sample was fabricated using the procedure shown in Fig. S1(a). First, an Ag layer with 180-nm thickness was deposited using an electron beam (e-beam) evaporator on double-side polished glass. A long slit for plasmon generation was created using the focused-ion-beam (FIB) method on the Ag layer. The slit length was 100 μm and the width was 135 nm (Fig. S1(b)). After slit fabrication, a thin dielectric layer with 50-nm thickness was deposited, composed of a mixture of poly(methyl methacrylate) (PMMA) and thinner, by spin-coating at 3,000 rpm for 40 s. At this point, we examined a transmission of the sample to determine if the plasmon had been well-generated. Figure S1(c) is a transmission image obtained by experiment and we can confirm that the long slit is well-defined and the PMMA layer is well-deposited on the metal layer. Then, an Ag layer with 300-nm thickness was deposited by e-beam evaporation to create the metal-insulator-metal (MIM) guide structure and slot antenna formation. The slot antenna was also fabricated by FIB, and the slot depth was gently tuned so as to not penetrate the MIM structure completely. The scanning electron microscope (SEM) image in Fig. S1(d) shows the FIB milling depth depending on the ion beam dose. We chose 300 nm as the slot depth.

**2. Dipole coupling measurement**

The dipole coupling experiment was conducted in cases with varying distances between the slit and slot antenna. As shown in Fig. 2(b), samples with separations of 1, 2, 4, 6, 8, and 10 μm were fabricated, but only the coupling image of the sample with 4-μm distance is shown in the main text. In this supplement, all of the coupling images, with both parallel and vertical geometry, are given in Fig. S2. As noted in the main text, we can clearly see that the guided plasmon is coupled to the slot parallel to the slit direction. As the distance increases, the coupling intensity decreases because of propagation loss. Using these experimental data, we were able to calculate the propagation length and compare the results with those obtained by the finite-difference time-domain (FDTD) simulation in Fig. 3. The transmitted intensity was integrated and normalized to the background intensity. In Fig. S2(b), the white circle corresponds to the integrated intensity captured by the MATLAB program and the intensity integrated in the red circle was used as the normalization intensity in the calculation. Then, the experimental propagation length was drawn in the graph shown in Fig. S2(c) for comparison. In fitting process of simulation results, we used the function of “*y = A_1_∙exp(-x/t_1_)*”, where *A_1_* and *t_1_* are fitting parameters. In experiment, *A_1_* and *t_1_* correspond to 32413.24(±4363.29) and 3.04(±0.60). In this case, because the intensity is not normalized by incident intensity, the number of *A_1_* is fitted as large number. In 3D FDTD simulation, *A_1_* and *t_1_* correspond to 0.00153(±4.04E-4) and 2.64 (±2.01). The error of propagation length is too large to adapt it. These large errors of 3D FDTD and experimental data come from the sinusoidal oscillation of output power in MIM plasmonic guide. The Febry-Perot mode between the slit trench and slot antenna makes this oscillation. 3D FDTD simulation and experimental data show this phenomenon exactly. Therefore, to make the propagation length confirmed in our experiment, we also calculated analytical propagation length.^1^ This MIM structure supports antisymmetric plasmonic waveguide mode, and has a propagation length of 2.9 μm in case of dielectric medium with 80 nm. (In case of dielectric medium with 50 nm, the propagation length is 2.0 μm. From this analysis, we found out that real thickness of PMMA layer is around 80 nm.) Therefore, we adapted the propagation length of 3 μm in maintext.

**3. Transmission images depending on polarization**

To confirm the coupling between the guided plasmon and resonance mode of the single-slot antenna, a polarization-dependent coupling experiment was executed. We used a slot antenna oriented at -45° to the slit, as represented schematically in Fig. S3(a), and changed the polarization direction while measuring the transmission image. The incident light polarization was controlled by a dichroic polarization filter located before the sample and another polarization filter was added before the charge-coupled device (CCD) detector. The transmitted light was measured depending on the polarization of the output light. From the results it is apparent that, when the incident light polarization and the direction of the measuring polarization filter are vertical to the slit and slot direction (V-V in the figure), we can see the transmitted light clearly, as shown in Fig. S3(b). If we rotate the polarization direction in the detection section by 90° (parallel to the slot), we cannot observe any signal, because the radiation from the single slot antenna is polarized in a direction vertical to the slot (V-P in Fig. S3(c)).^2^ Then, if we change the polarization of incident light by 90° (parallel to the slit), a plasmon cannot be generated and we cannot see the transmitted light from the slot (P-V in Fig. S3(d)). This proves that the transmitted light comes from coupling of the guided plasmon directly. If the incident light penetrates the bottom metal and couples to the slot antenna directly, we should see transmitted light in the case of P-V, because the incident light couples to the slot antenna in a ratio of *sin(45°)*. However, we cannot detect any signal in this case, as shown in Fig. S3(d). Therefore, we can determine that the transmitted output comes from the guided plasmon only in the coupling experiment. (See the coupling experiment movie demonstrating the rotating incident polarization direction.)

**4. Optical measurement setup**

The real-space and Fourier-space images were measured simultaneously in one setup, as shown in Fig. S4. To obtain the Fourier-space image, we used a lens placed before an electron-multiplying CCD (emCCD) detector. In Fig. S4, the real-space plane is the iris position and, if a detector were positioned at the focal plane of the lens (*f*), we could easily obtain the Fourier-space image.^3^ Then, the real-space image could be captured by switching the mirror. To convert the Fourier-space image into a real-space image, we used another lens with the same *f*. Positioning the CCD detector at a distance of *2f* from the first lens, we could obtain a real-space image again.

**5. Apodization process**

Objective with large numerical aperture has anisotropic photon intensity distribution, thus correct apodization should be performed in order to compare the simulated with the measured Fourier-space images.^2, 4^ This apodization was executed over the whole Fourier-space images. To get the Fourier-space image at the hemisphere with 1-m radius, we took the 2-dimensional (2D) intensity distribution in the back focal plane of the objective lens with NA = 0.95 using emCCD. The 2D intensity distribution and the Fourier-space image are related by the well-known relation

$I(\theta,\varphi)= I(k_{x},k_{y}) \cos(\theta)$,

where $k_{x}=KR\sin(\theta)\cos(\varphi)$ and $k_{y}=KR\sin(\theta)\cos(\varphi)$. Here, the cosine factor is the apodization factor, and *K* is a calibration constant related to the magnification of the imaging system, which is obtained by fitting the grating diffraction orders with known periods. Grating patterns obtained in our emCCD are shown in Fig. S5(b). Using the relation above, we obtained the Fourier-space image at the hemisphere from the 2D image captured by emCCD.

**6. Calculation of coupling efficiency between MIM plasmonic guide and slot antenna.**

To find out the coupling efficiency between MIM plasmonic guide and slot antenna, we executed analytical calculation. Figure S6 shows the schematics used in analysis. The incident light with intensity *I_0_* from back side of sample generates surface plasmon at the position of slit. We denote the coupling efficiency between incident light and slit structure as *C_1_*. The generated surface plasmon forms antisymmetric transvers magnetic (TM) waveguide mode. The intensity of guide mode decays exponentially because of metallic loss. When the propagating mode meets with slot antenna, it converted into light with intensity I_2_ and radiated into upside of top metal layer through slot antenna. We denote the efficiency of this conversion as *C_2_*, and this is the coupling efficiency of slot antenna (nominated as C in maintext). Therefore, relation between *I_0_* and *I_2_* can be written as$I_{2}=C_{2}e^{-x/L}C_{1}I_{0} \Rightarrow\frac{I_{2}}{I_{0}}=C_{2}C_{1}e^{-x/L}$. From this function, we know that the output intensity form has the first-order exponential decay function. When *I_2_* is normalized by incident intensity *I_0_*, the fraction of function corresponds to total coupling efficiency of the structure including slit, MIM plasmonic guide, and slot antenna.

In 3D FDTD simulation, we found out that simulated total efficiency *C_1_C_2_* is around 0.15 % by fitting process described the above supplementary section 2. We used measured output power located by 100 nm apart from the top metal surface because of propagating surface plasmon on top metal. To find out the coupling efficiency between slot antenna and MIM plasmonic guide, we used input intensity simulated at the position by 100 nm apart from slot antenna edge to exclude scattering effect of edge. Because this incident intensity from guiding structure to slot antenna cannot be measured in experiment, we calculated this efficiency by the help of FDTD simulation. To remove position dependence of input power, we averaged intensity with one period of 664 nm wavelength. The efficiency *C_2_* (nominated as *C* in maintext) is calculated as 19.23 % in FDTD simulation. This is very similar with coupling efficiency obtained in structure with rod antenna and dielectric waveguide.^5^ From two results of total efficiency *C_1_C_2_* and coupling efficiency *C_2_* of slot antenna, we found out that coupling efficiency of slit with 135 nm width has around 0.78 % at the wavelength of 664 nm.^6^

**7. Analysis of groove structure**

**7-1. Mode formation**

As like slot-slot interaction described in reference 4, mode excitation inside a feed slot at resonance should work regardless of the existence of an adjacent slot or groove. In order to check this, we plot the calculated electric field intensities of the slot and groove upon a polarized incident laser. As shown in Fig. S7(a), strong electromagnetic mode indeed forms inside the feed slot even with an adjacent groove in all cases. In this case, metal thickness, feed length, and groove depth (δ) are fixed as 300 nm, 180 nm, and 100 nm same to experimental condition. When 100 nm distance between slot and groove (D) and groove length (L) > 200 nm, weak mode formation in the groove can be observed suggesting that coupling between the slot and groove emerges. From simulated far-field radiation pattern of Fig. S7(b), we know that this coupled mode formation would result in strong directional radiation.

**7-2. Far-Field radiation patterns**

To distinguish the role of director or reflector, we draw radiation patterns in different parameters of distance and groove length. The structure with slot and groove is located on the x-y plane in the spherical coordinate system. Figure S7(b) shows the intensity plots of the polarization filtered radiation in upper hemisphere with r = 1 m using near-to-far-field transformation analysis of the FDTD results. As like conventional Yagi-Uda antenna, we see that groove shorter than the feed slot length works as weak director, while groove longer than the feed slot works as a reflector given fixed groove depth of 100 nm and distance shorter than 180 nm. In region with distance longer than 180 nm, the role of groove as director gets weaker. Additionally, we also found out that this trend depends on the groove depth. The dependence of groove depth effect will be discussed in later section.

**7-3. Charge distribution and Phase difference**

In order to understand the nature of the coupling between slot and groove, we analyze the charge density distribution and phase of combined structure. Form the charge density distribution of Fig. S7(c), we know that the coupling between slot and groove is inductive in condition of director (for example, the charge sign in condition with D = 100 nm and L = 200 nm), while capacitive in condition of reflector (for example, the charge sign in condition with D = 100 nm and L = 300 nm). These results are opposite to the coupling in multiple-rod based RF antenna. It implies Babinet’s principle, which states that the electric field and the magnetic field are exchanged for a pair of complementary structures.^7^ We see that the coupling is following the trend of the directional radiation exactly obtained in Fig. S7(b).

However, just looking at the charge configuration is not enough to find optimal conditions for reflector and director. Therefore, we analyze the phase distribution of the x-component of the electric field, as shown in Fig. S7(d). Compared to the x-component, the y- and z-components are very weakly coupled to slot and groove.^8^ Therefore, we chose the x-component of the electric field at the center of each slot and groove to define the phase. We find that the phases inside the slot and groove are almost uniform, and therefore, we can define a single-valued phase by taking the value at the center of slot and groove. The uniformity of the phase inside slot and groove suggests that each element can be regarded as a single dipole object.

From calculated FB ratio contour map (Fig. S7(e)) and phase distribution (Fig. S7(f)), we have found that the phase difference, which is defined as the phase of the groove minus the phase of feed, has a close relationship with directionality. For a fixed length of groove element, FB ratio gets increased as the distance between slot and groove gets decreased. The interaction between slot and groove happens by the help of plasmon, and it seems that the interaction becomes decayed exponentially. For a fixed distance between slot and groove, as the phase difference gets to 90°, the FB ratio gets increased. We find that the phase difference with 90° shows right directionality with large FB ration, suggesting constructive interference of electromagnetic waves to right direction.^2^ In the experiment, we fixed the distance as 120 nm. This distance is the minimum one because the proximity effect prevents to making slot and groove with narrow width by FIB milling. We chose the length of groove as 280nm. In FDTD simulation, the FB ratio in this structure has about 8.51. In experiment, we obtained FB ration of 7.38.

**7-4. Groove depth effect**

To understand the groove depth effect on directionality, we calculated two cases of depth with 100 nm and 50 nm. As like the above analysis, mode formation, far-field radiation pattern, charge distribution, and phase difference were calculated and compared. From the far-field radiation pattern in the Fig. S7(g) of groove with 50 nm depth, we know that the groove does not work as reflector, but only director in condition with the distance shorter than 200 nm and length shorter than 500 nm. We can see the far-field radiation pattern and phase difference map of both groove with 50 nm depth in Fig. S7(g) and (h).

**References**

1. Dionne, J. A., Sweatlock, L. A., Atwater, H. A. & Polman, A. Plasmon slot waveguides: Towards chip-scale propagation with subwavelength-scale localization. *Phys. Rev. B* **73,** 035407 (2006)

2. Kim, J. *et al.* Babinet-inverted optical Yagi–Uda antenna for unidirectional radiation to free space. *Nano Lett.* **14,** 3072–3078 (2014).

3. Saleh, B. E. A. & Teich, M.C. in *Fundamentals of photonics 1st edn,* Ch. 4, 124-127 (Wiley, 1991)

4. Lee, K. G. *et al.* A planar dielectric antenna for directional single-photon emission and near-unity collection efficiency. *Nat. Photon.* **5,** 166-169 (2011).

5. Arango, F. B., Kwadrin, A. & Koenderink, A. F. Plasmonic antennas hybridized with dielectric waveguides. *ACS Nano* **6,** 10156-10167 (2012).

6. Chen, J., Li, Z., Yue, S. & Gong, Q. Efficient unidirectional generation of surface plasmon polaritons with asymmetric single-nanoslit. *Appl. Phys. Lett.* **97,** 041113 (2010)

7. Balanis, C. A. in *Antenna theory: analysis and design*, *3rd edn,* Ch. 12, 697-701 (Wiley-Interscience, 2005).

8. Gordon, R. & Brolo, A. *Opt. Express* **13,** 1933–1938 (2005).

**Figures & Figure Captions**

**
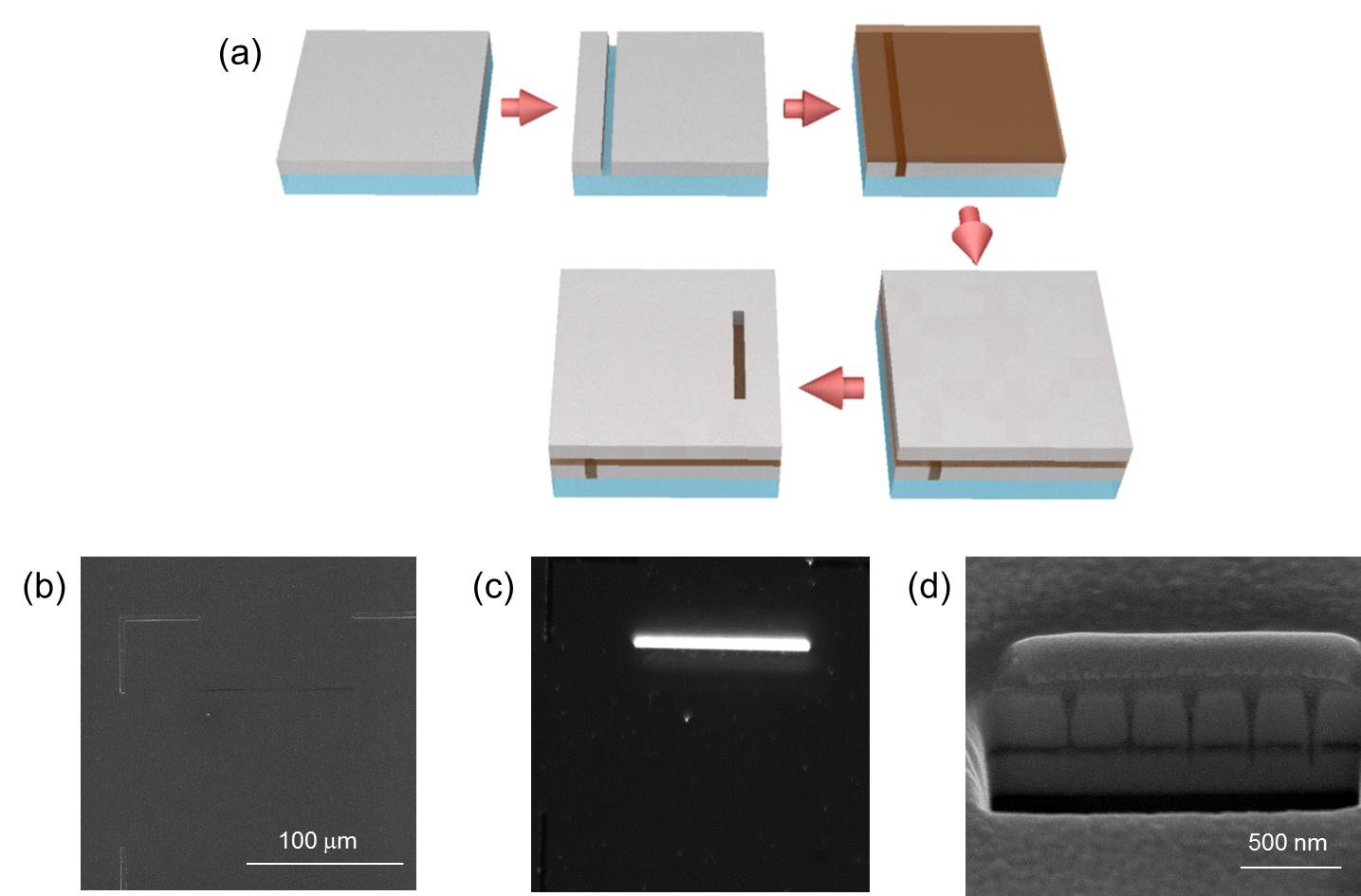
**

**Figure S1. Fabrication procedure** (a) Sample fabrication procedures were as follows: An Ag layer with 180-nm thickness was deposited by e-beam evaporator and a long slit with 135-nm line-width and 100-μm length was milled using the FIB method. A PMMA and thinner mixture was spin-coated at 3,000 rpm for 40 s and the PMMA thickness was adjusted to 50 nm for plasmon-guiding only, as the PMMA layer is a guiding layer in the MIM structure. An Ag layer with 300-nm thickness was also deposited by e-beam evaporator on the PMMA layer. The slot-type antenna was fabricated on the top metal layer using the FIB method. (b) SEM image of slit with 100-mm length and 135-nm width. (c) Transmission image after PMMA coating. (d) SEM image of depth tuning for various ion beam doses. The dose increased from left to right and the depth increased simultaneously.

**
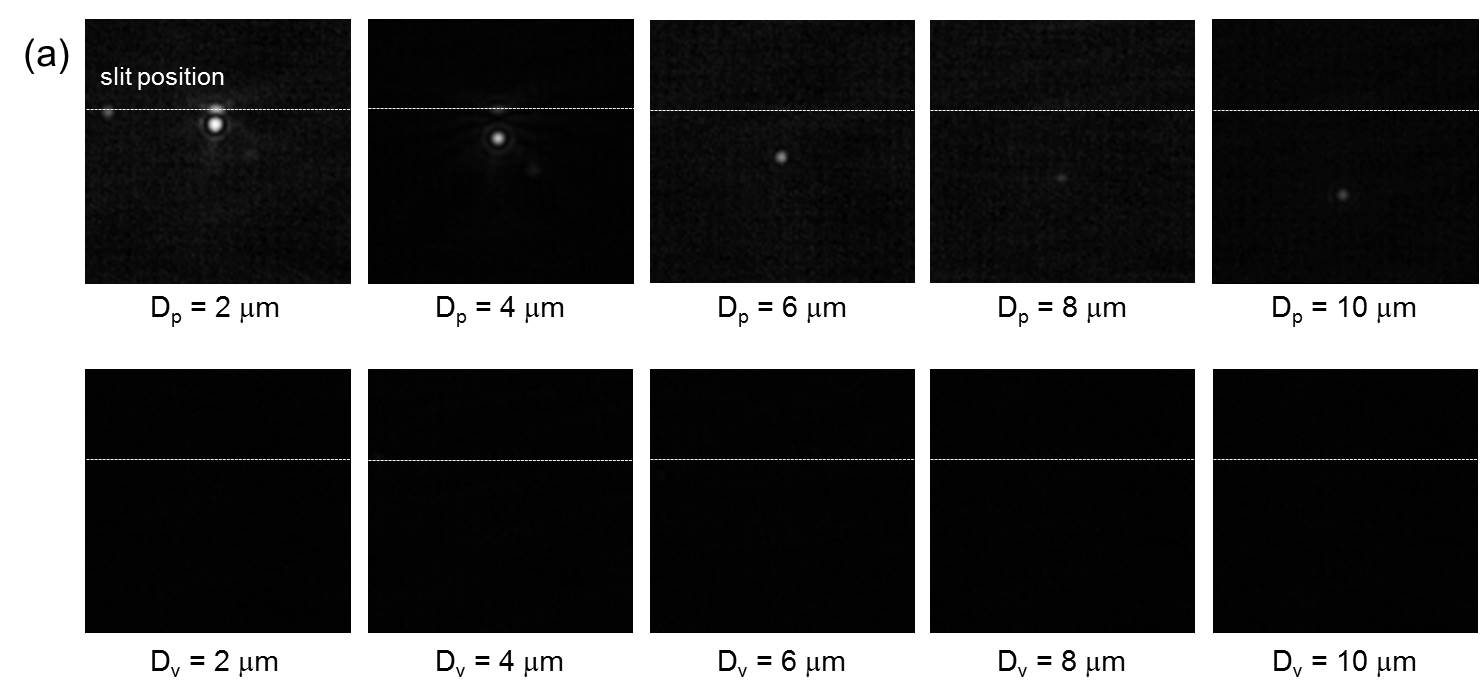
**

**
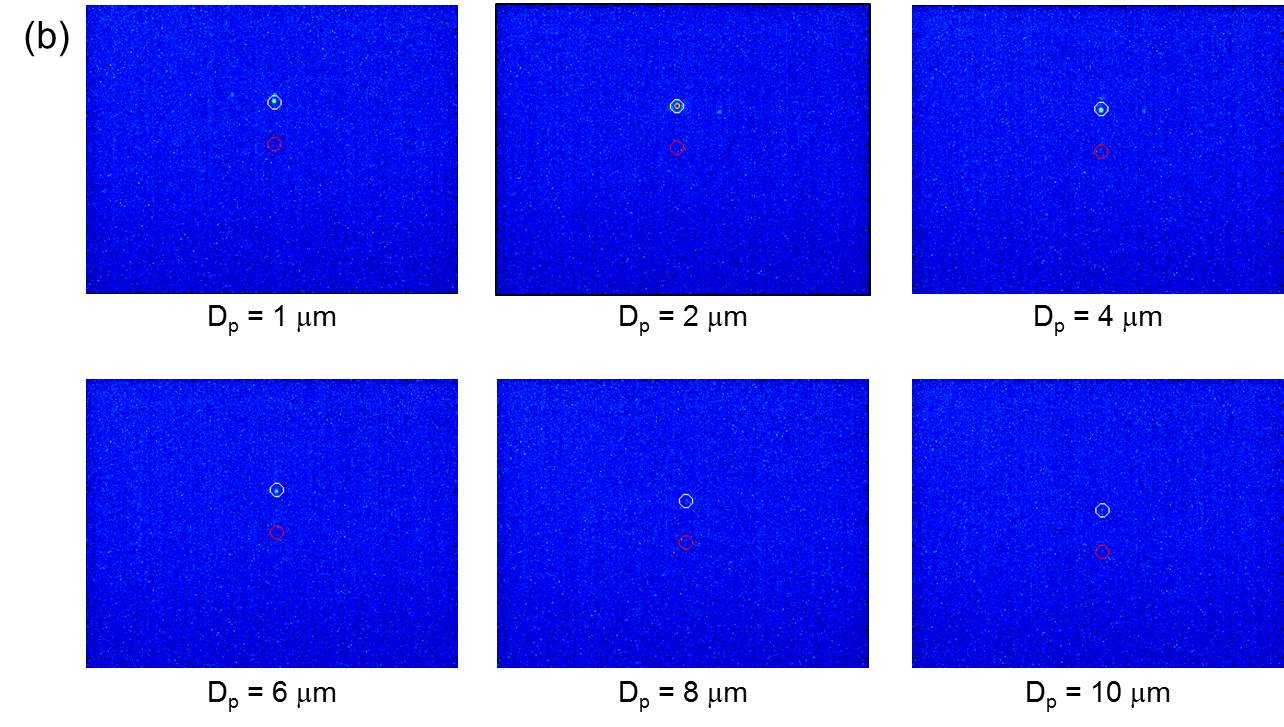
**

**
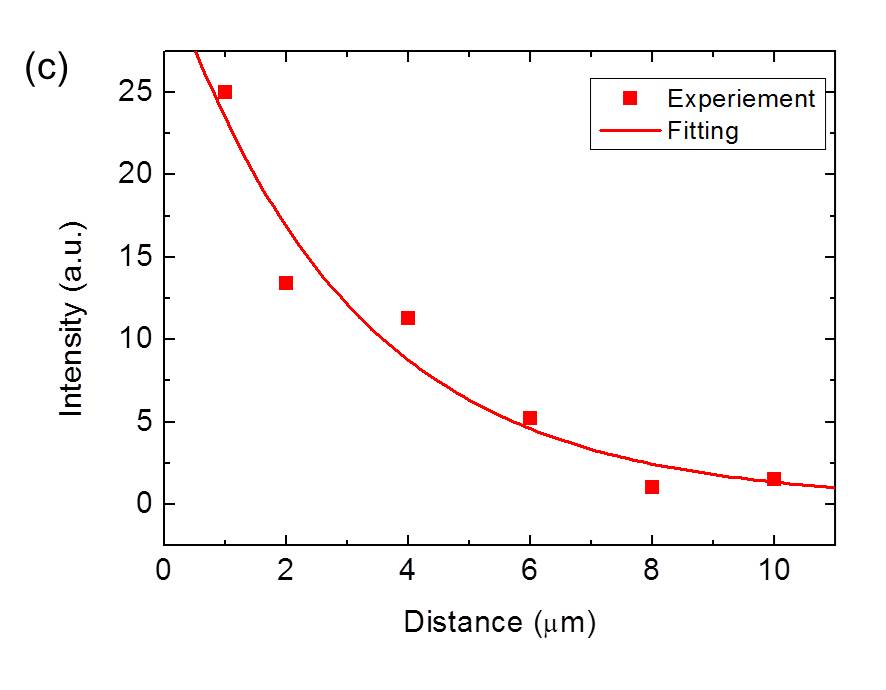
**

**Figure S2. Dipole coupling measurement and propagation length** (a) Transmitted light images with varying distances and polarization. In the first line of images, the slot direction is parallel to the slit and, in the bottom line, the slot direction is vertical to the slit. The white dashed line represents the slit position. The plasmon guide mode is coupled to the slot antenna mode in the parallel slot case only. The output light intensity decreases as the distance increases as a result of propagation loss. (b) Images converted by Matlab program for intensity integration. The intensity within the region indicated by the white circle was integrated, and we used intensity integration of the area within the red circle as the background signal for normalization. (c) Integrated intensities plotted as functions of distance. The red rectangle shows the integrated intensity and the red line is a curve fit as the first-order exponential function. We determined that the propagation length of our MIM structure is 3.0 μm.

**
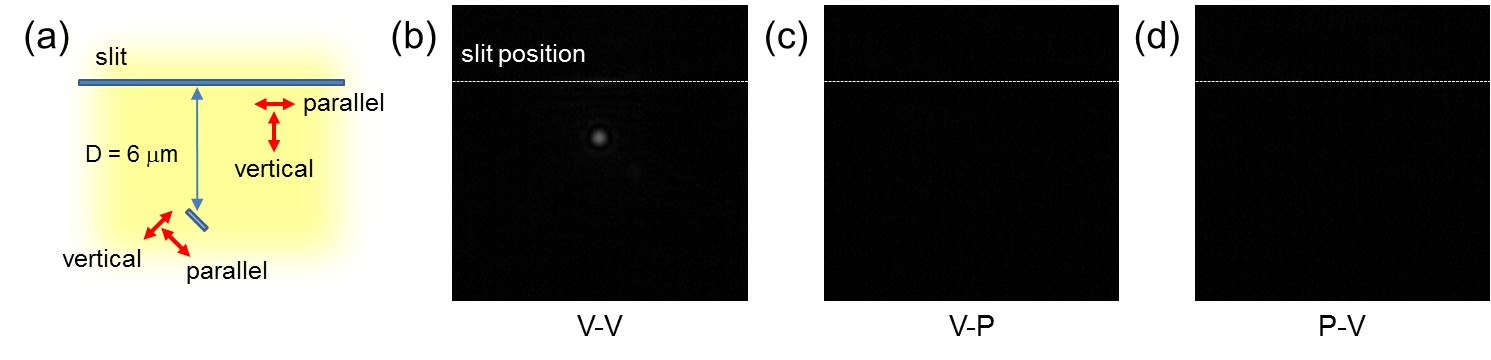
**

**Figure S3. Polarization-dependent coupling measurement** (a) Polarization-rotation measurement sample schematics. The distance between the slit and slot is 6 μm and the slot antenna direction is rotated by 45° relative to the slit direction. The red arrows show the polarization directions of each slit and slot. (b) When the incident and detection polarizations are vertical to the slit and slot simultaneously (V-V), we see a bright transmitted output signal from the slot antenna. (c) When the incident polarization is vertical to the slit and the detection polarization is parallel to the slot (V-P), we cannot observe any signal because the slot antenna supports vertical polarization only. (d) When the incident polarization is parallel to the slit and the detection polarization is vertical to the slot (P-V), we cannot observe any signal because the slit cannot generate a plasmon. This result proves directly that the transmitted light comes from a plasmon guided by the MIM structure.

**
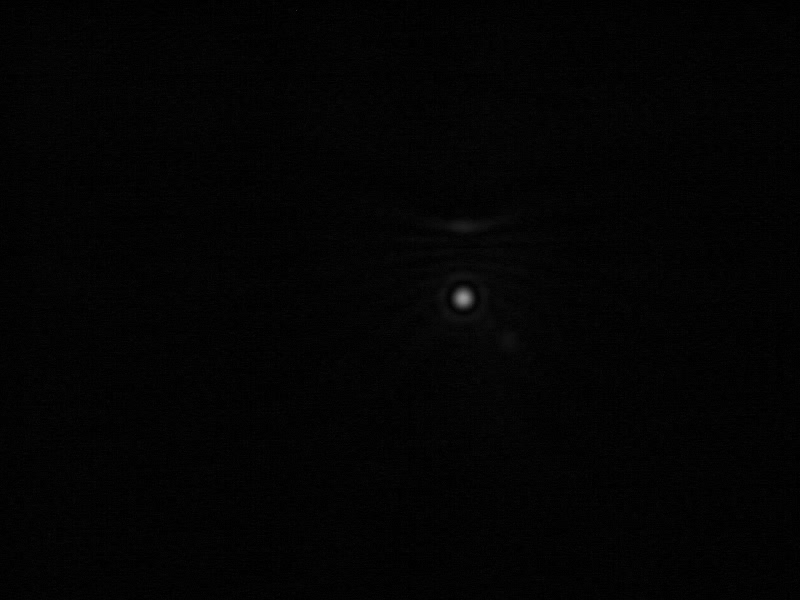
**

**Movie S1.** While rotating the detection polarization from the vertical to the parallel direction, the coupling intensity decreases and disappears. When the detection polarization direction is rotated by 360°, we observe bright output light from the slot again.

**
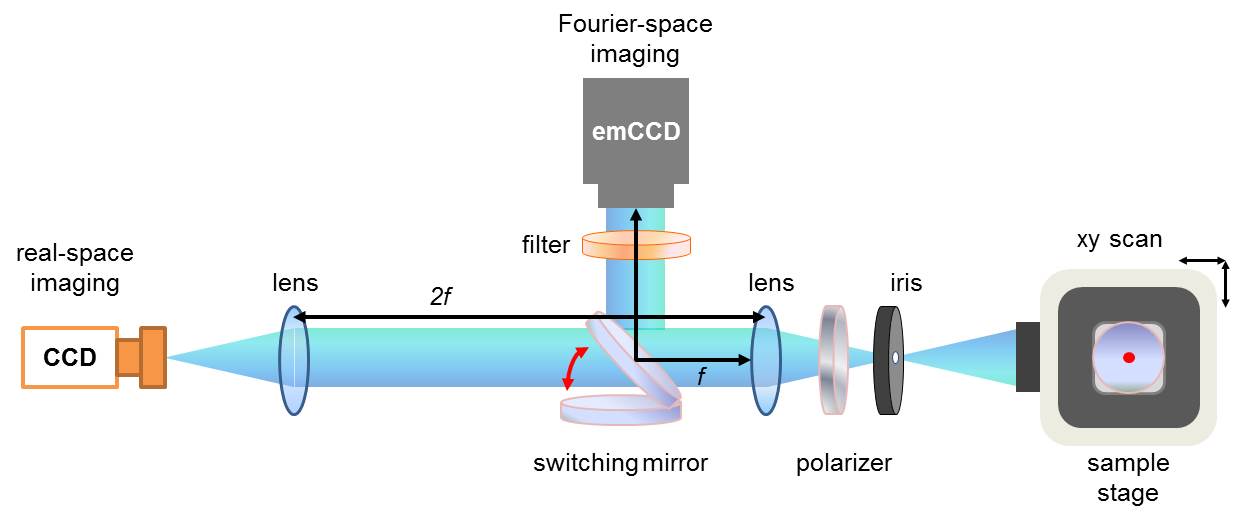
**

**Figure S4. Fourier-space and real-space measurement setup.** Fourier-space and real-space image setup schematics. When the detection device is positioned at the focal length, *f*, of the lens, we can obtain a Fourier-space image.


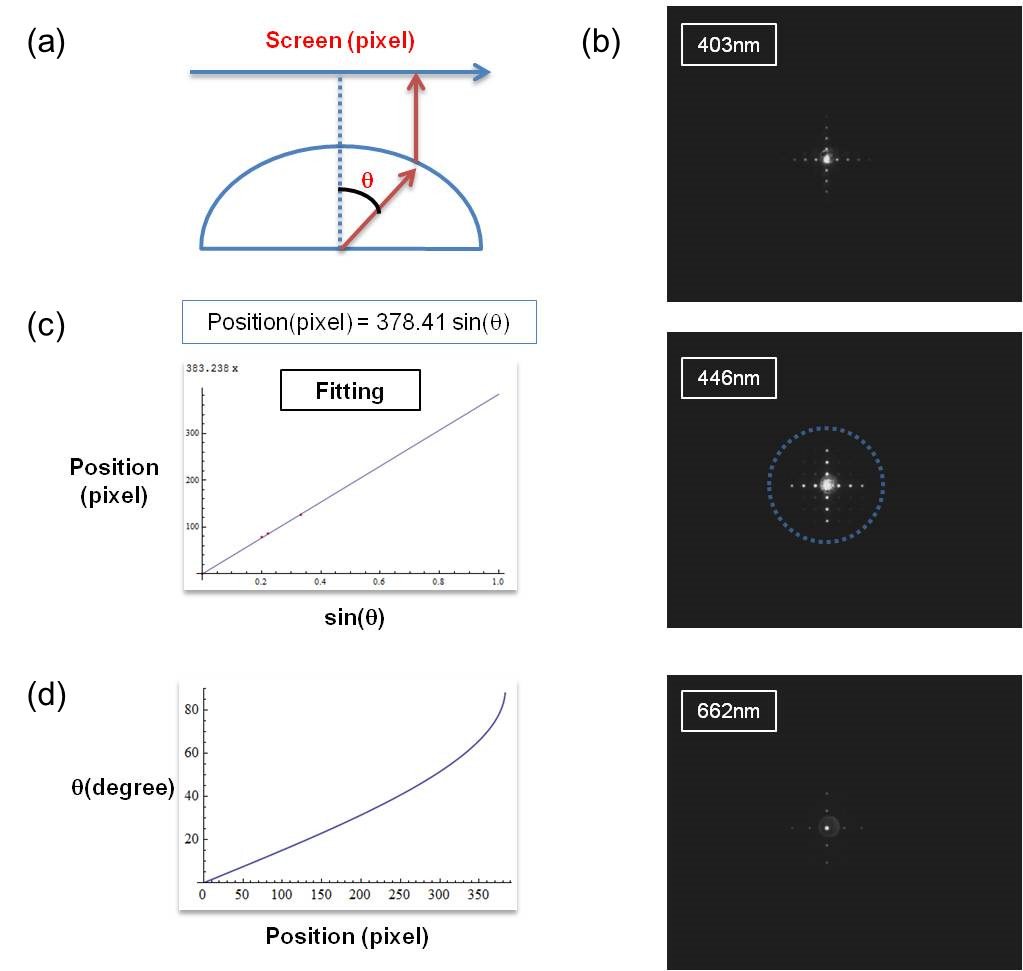


**Figure S5. Apodization process.** (a) Schematic of the apodization between a 2D plane and a hemisphere. (b) Diffraction patterns of the gratings obtained by emCCD for different laser sources. (c) Graph of the fit for the calibration constant *K* from the diffraction patterns. (d) Obtained apodization relation between the position in the 2D image and the angle of the hemisphere.


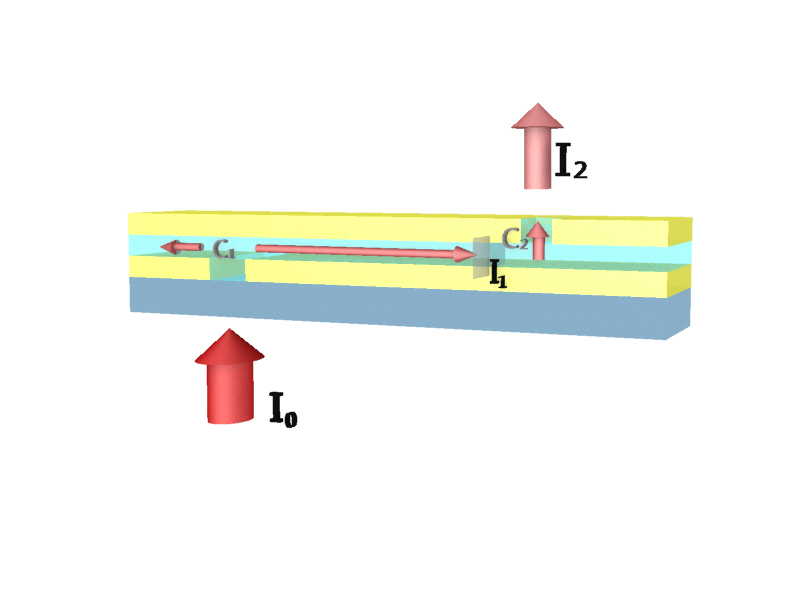


**Figure S6. Model of coupling efficiency calculation.** The incident light with *I_0_* is coupled with plasmon at slit position. The coupling efficiency is denoted as *C_1_*. The propagated plasmon is decayed exponentially, and coupled with output light of *I_2_* at slot antenna position. This coupling efficiency is denoted as *C_2_*.


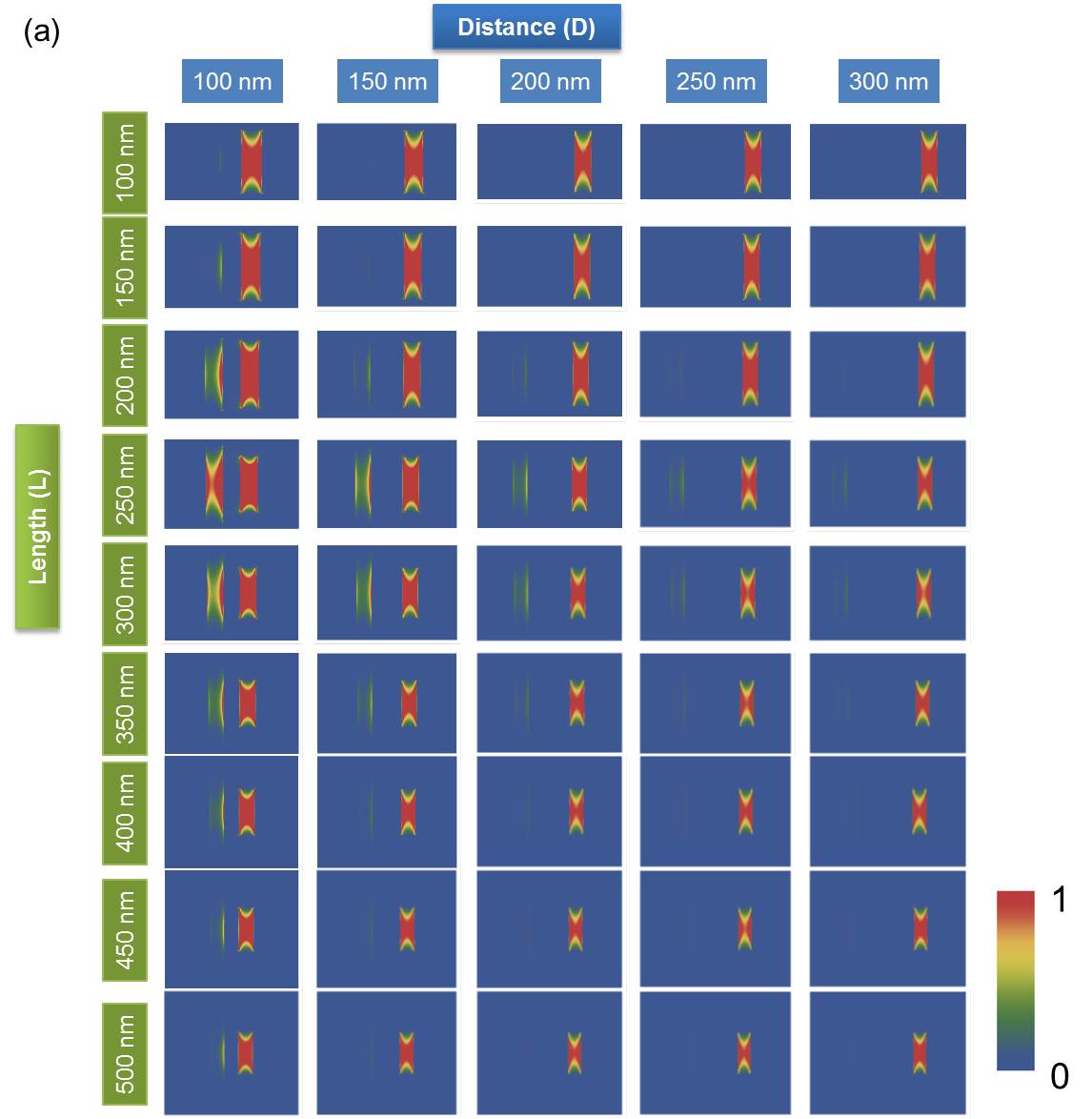


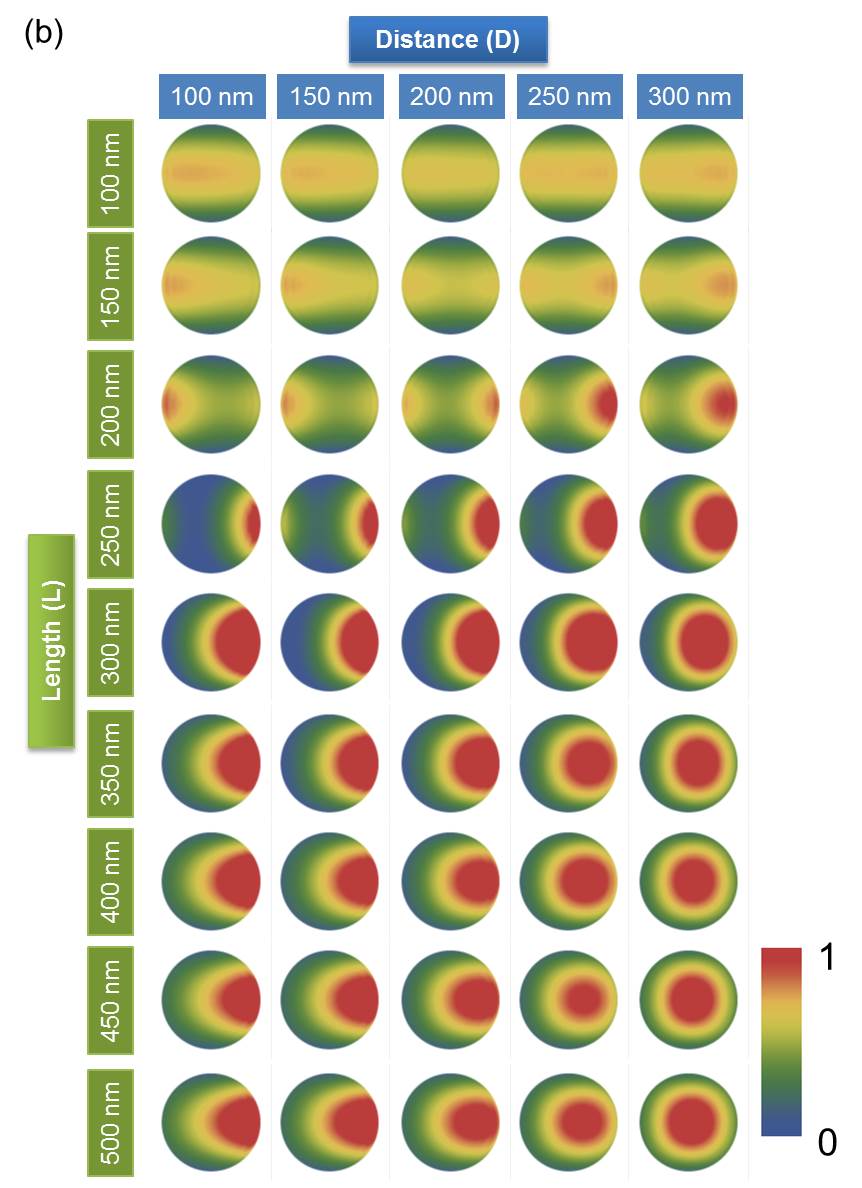


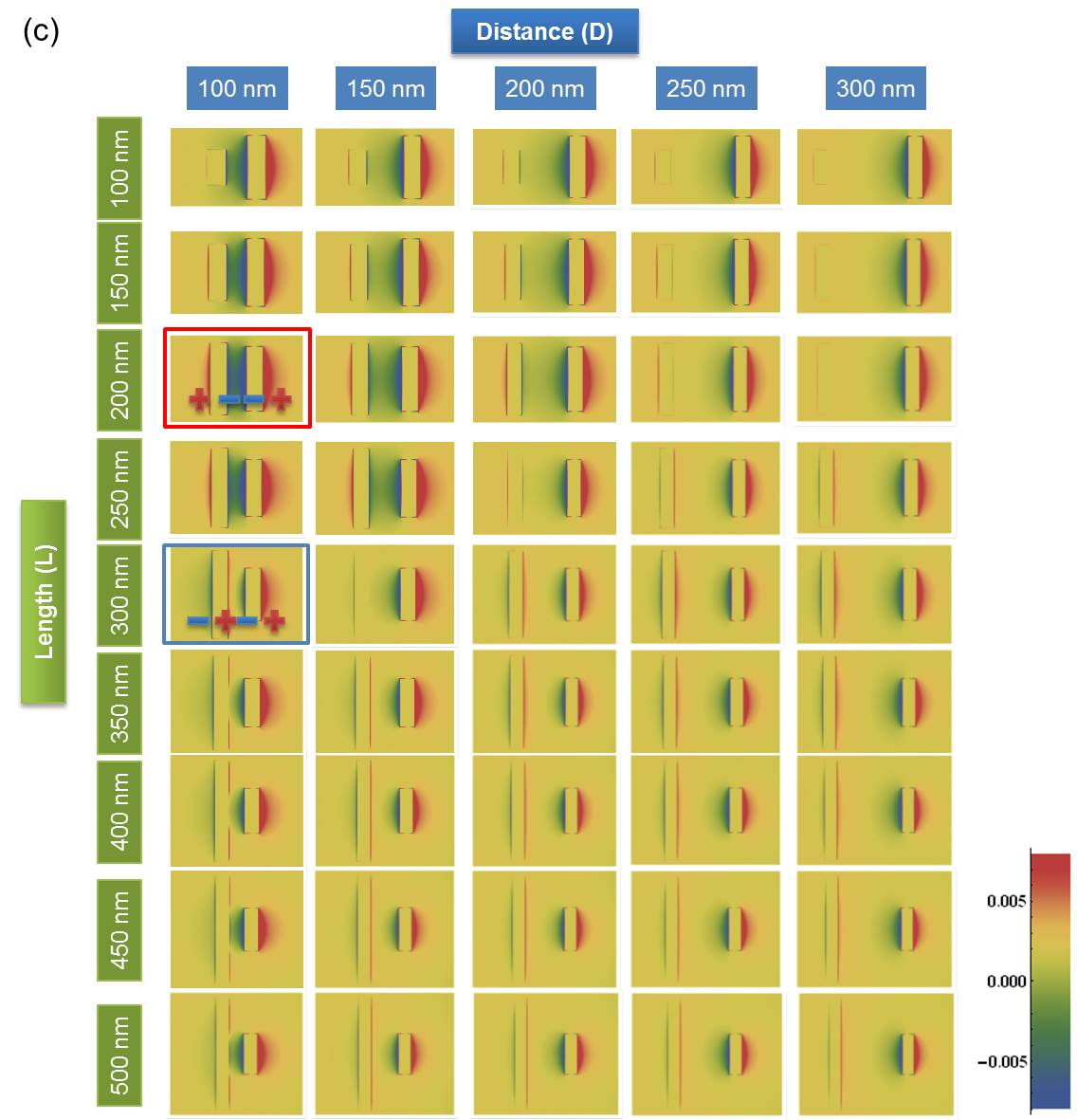


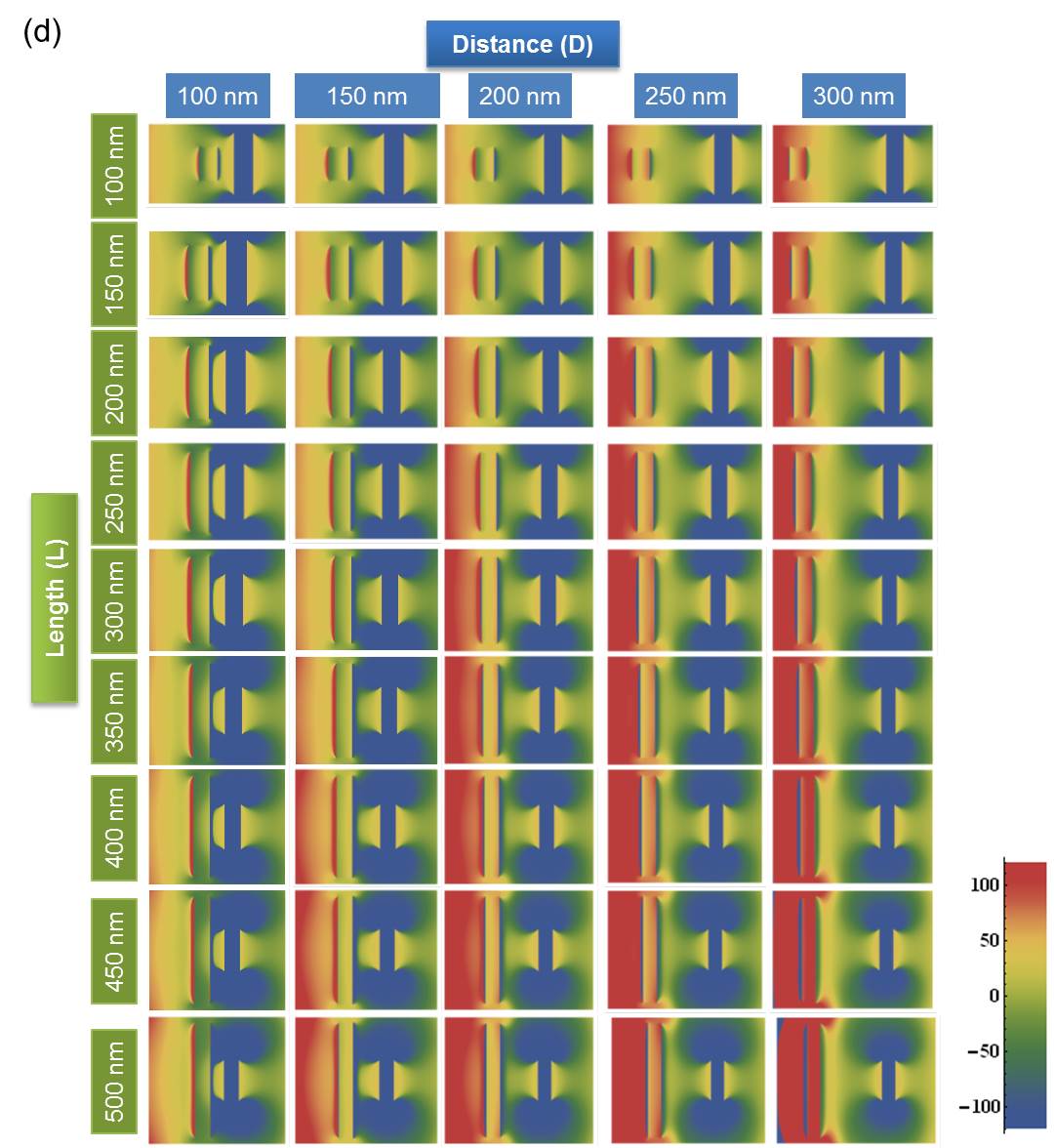


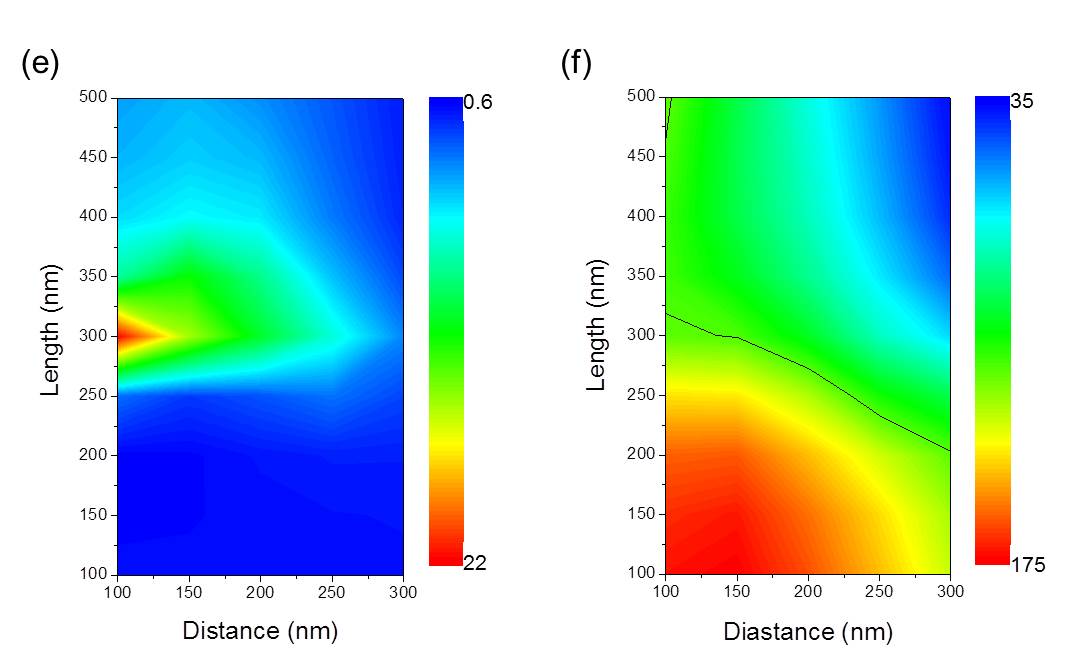


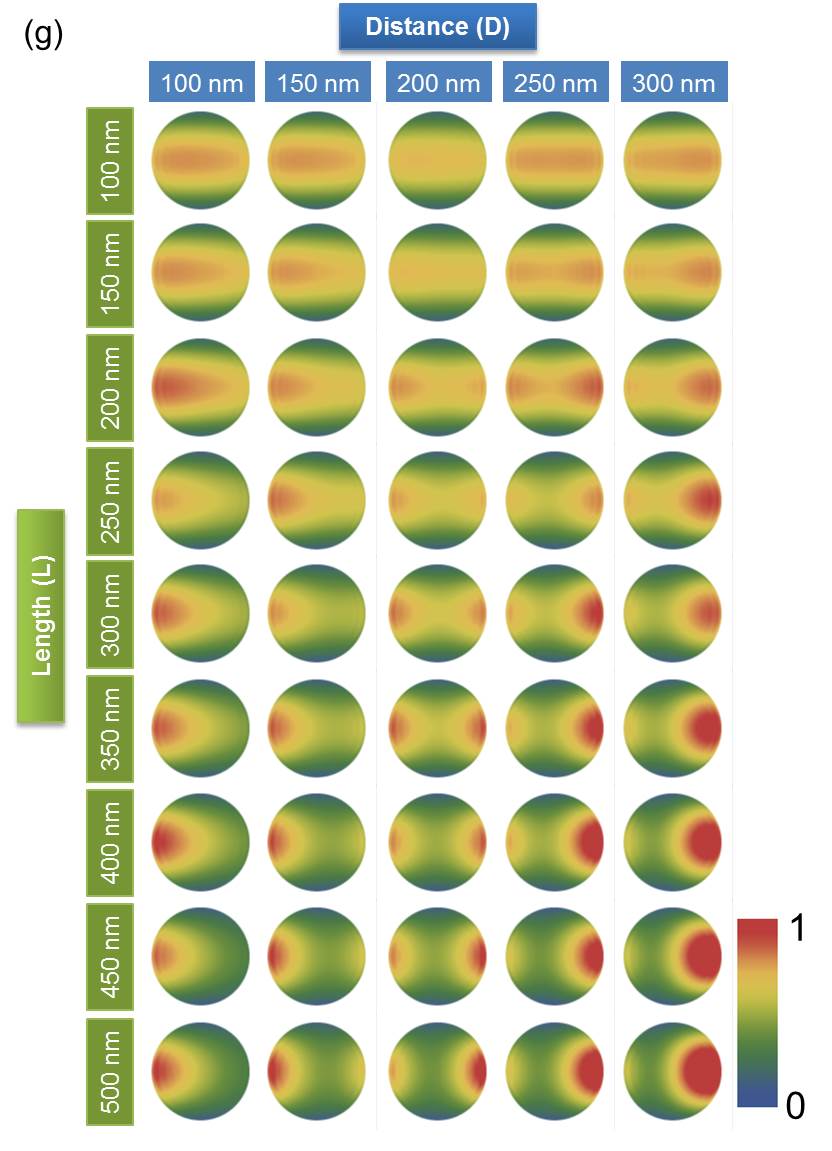


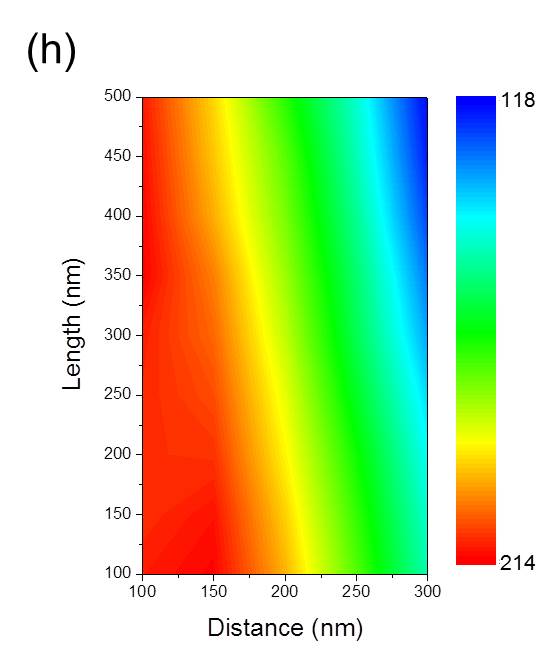


**Figure S7. Analysis of slot and groove structure** (a) Map of the normalized electric-field (E-field) intensity of slot and groove structure for different lengths (L) and distances (D). The depth of groove is fixed as 100 nm. When the slot and groove structure is irradiated by x-polarized plane wave at 664 nm, fundamental electromagnetic mode is formed inside the slot regardless of the adjacent groove element. (b) Map of the normalized far-field intensities in the upper hemisphere with a 1-m radius for various L and D values. (c) Map of the charge distribution for slot and groove structure with different L and D. The good reflector and director configurations have capacitive and inductive charge couplings between the two elements, respectively. The red (blue) box corresponds to the director (reflector). This coupling feature is exactly opposite to the case of a multiple-rod-based antenna, as expected from Babinet’s principle. (d) Map of the phase distribution of the x-component of the E-field. The phase is almost uniform inside a slot and a groove, which enables us to define a single-valued phase measured at the center of each element. (e) Contour plots of the FB ratios and (f) phase differences. We found that the phase difference and the FB ratio have a close relationship. We observe that the good reflector condition overlaps with the 90° phase difference line (black line in Fig. S7(f)). (g) Map of the normalized far-field intensities for slot and groove with 50 nm depth. (h) Contour plot of phase difference for slot and groove with 50 nm depth.
